# Supplementary material for: Farm diversification as a potential success factor for small-scale farmers constrained by COVID-related lockdown. Contributions from a survey conducted in four European countries during the first wave of COVID-19
Source: PLoS One. 2021 May 21;16(5):e0251715. doi: 10.1371/journal.pone.0251715 (PMC8139471; doi:10.1371/journal.pone.0251715)
Supplement: S1 Table — (DOCX) [file pone.0251715.s001.docx]

# S1 Table. Country-specific descriptive statistics (Estonia).

| Variable | N | Frequency (%) | Mean | SD | Min | Max |
| --- | --- | --- | --- | --- | --- | --- |
| Increase in sales | 52 | - | 0.307 | 0.466 | 0 | 1 |
| Channel diversification | 52 | - | 3.500 | 2.262 | 0 | 9 |
| Number of channels: 0 | 3 | 5.7 | - | - | - | - |
| Number of channels: 1 | 6 | 11.5 | - | - | - | - |
| Number of channels: 2 | 12 | 23.1 | - | - | - | - |
| Number of channels: 3 | 9 | 17.4 | - | - | - | - |
| Number of channels: 4-6 | 15 | 28.8 | - | - | - | - |
| Number of channels: 7 or more | 7 | 13.5 | - | - | - | - |
| Product diversification | 52 | - | 1.326 | 0.584 | 1 | 3 |
| Number of products: 1 | 43 | 82.7 | - | - | - | - |
| Number of products: 2 | 7 | 13.5 | - | - | - | - |
| Number of products: 3 | 2 | 3.8 | - | - | - | - |
| Number of products: 4 | 0 | 0 | - | - | - | - |
| Number of products: 5 or more | 0 | 0 | - | - | - | - |
| Income | 52 | - | 3.384 | 1.510 | 1 | 5 |
| Below €5,000 | 7 | 13.5 | - | - | - | - |
| €5,000 - €15,000 | 11 | 21.2 | - | - | - | - |
| €15,000 - €30,000 | 9 | 17.3 | - | - | - | - |
| €30,000 - €50,000 | 5 | 9.6 | - | - | - | - |
| More than €50,000 | 20 | 38.4 | - | - | - | - |
| Fruits and vegetables | 52 | - | 0.170 | 0.380 | 0 | 1 |
| Egg or poultry | 52 | - | 0.120 | 0.320 | 0 | 1 |
| Meat | 52 | - | 0.120 | 0.320 | 0 | 1 |
| Milk and dairy | 52 | - | 0.150 | 0.360 | 0 | 1 |
| Honey | 52 | - | 0.150 | 0.360 | 0 | 1 |
| Bakery products | 52 | - | 0.130 | 0.340 | 0 | 1 |
| Herbs | 52 | - | 0.130 | 0.340 | 0 | 1 |
| Wine and grapes | 52 | - | 0.380 | 0.190 | 0 | 1 |
