# Supplementary material for: Farm diversification as a potential success factor for small-scale farmers constrained by COVID-related lockdown. Contributions from a survey conducted in four European countries during the first wave of COVID-19
Source: PLoS One. 2021 May 21;16(5):e0251715. doi: 10.1371/journal.pone.0251715 (PMC8139471; doi:10.1371/journal.pone.0251715)
Supplement: S2 Table — (DOCX) [file pone.0251715.s002.docx]

# S2 Table. Country-specific descriptive statistics (Hungary).

| Variable | N | Frequency (%) | Mean | SD | Min | Max |
| --- | --- | --- | --- | --- | --- | --- |
| Increase in sales | 136 | - | 0.095 | 0.295 | 0 | 1 |
| Channel diversification | 136 | - | 2.279 | 1.737 | 0 | 8 |
| Number of channels: 0 | 13 | 9.6 | - | - | - | - |
| Number of channels: 1 | 40 | 29.4 | - | - | - | - |
| Number of channels: 2 | 35 | 25.7 | - | - | - | - |
| Number of channels: 3 | 23 | 16.9 | - | - | - | - |
| Number of channels: 4-6 | 20 | 14.7 | - | - | - | - |
| Number of channels: 7 or more | 5 | 3.7 | - | - | - | - |
| Product diversification | 136 | - | 1.308 | 0.614 | 1 | 5 |
| Number of products: 1 | 107 | 78.7 | - | - | - | - |
| Number of products: 2 | 25 | 18.3 | - | - | - | - |
| Number of products: 3 | 3 | 2.3 | - | - | - | - |
| Number of products: 4 | 0 | 0.0 | - | - | - | - |
| Number of products: 5 or more | 1 | 0.7 | - | - | - | - |
| Income | 131 | - | 2.931 | 1.463 | 1 | 5 |
| Below €5.000 | 25 | 19.1 | - | - | - | - |
| €5.000 - €15.000 | 39 | 29.7 | - | - | - | - |
| €15.000 - €30.000 | 17 | 13.0 | - | - | - | - |
| €30.000 - €50.000 | 20 | 15.3 | - | - | - | - |
| More than €50.000 | 30 | 22.9 | - | - | - | - |
| Fruits and vegetables | 136 | - | 0.240 | 0.430 | 0 | 1 |
| Egg or poultry | 136 | - | 0.090 | 0.280 | 0 | 1 |
| Meat | 136 | - | 0.120 | 0.320 | 0 | 1 |
| Milk and dairy | 136 | - | 0.190 | 0.390 | 0 | 1 |
| Honey | 136 | - | 0.090 | 0.280 | 0 | 1 |
| Bakery products | 136 | - | 0.060 | 0.240 | 0 | 1 |
| Herbs | 136 | - | 0.110 | 0.310 | 0 | 1 |
| Wine and grapes | 136 | - | 0.070 | 0.260 | 0 | 1 |
