# Supplementary material for: Farm diversification as a potential success factor for small-scale farmers constrained by COVID-related lockdown. Contributions from a survey conducted in four European countries during the first wave of COVID-19
Source: PLoS One. 2021 May 21;16(5):e0251715. doi: 10.1371/journal.pone.0251715 (PMC8139471; doi:10.1371/journal.pone.0251715)
Supplement: S3 Table — (DOCX) [file pone.0251715.s003.docx]

# S3 Table. Country-specific descriptive statistics (Portugal).

| Variable | N | Frequency (%) | Mean | SD | Min | Max |
| --- | --- | --- | --- | --- | --- | --- |
| Increase in sales | 76 | - | 0.223 | 0.419 | 0 | 1 |
| Channel diversification | 76 | - | 3.000 | 2.508 | 0 | 12 |
| Number of channels: 0 | 7 | 9.2 | - | - | - | - |
| Number of channels: 1 | 16 | 21.1 | - | - | - | - |
| Number of channels: 2 | 21 | 27.6 | - | - | - | - |
| Number of channels: 3 | 8 | 10.5 | - | - | - | - |
| Number of channels: 4-6 | 18 | 23.7 | - | - | - | - |
| Number of channels: 7 or more | 6 | 7.9 | - | - | - | - |
| Product diversification | 76 | - | 1.289 | 0.708 | 1 | 9 |
| Number of products: 1 | 62 | 81.6 | - | - | - | - |
| Number of products: 2 | 8 | 10.5 | - | - | - | - |
| Number of products: 3 | 5 | 6.6 | - | - | - | - |
| Number of products: 4 | 0 | 0.0 | - | - | - | - |
| Number of products: 5 or more | 1 | 1.3 | - | - | - | - |
| Income | 61 | - | 3.540 | 1.576 | 1 | 5 |
| Below €5,000 | 10 | 16.4 | - | - | - | - |
| €5,000 - €15,000 | 10 | 16.4 | - | - | - | - |
| €15,000 - €30,000 | 5 | 8.2 | - | - | - | - |
| €30,000 - €50,000 | 9 | 14.8 | - | - | - | - |
| More than €50,000 | 27 | 44.2 | - | - | - | - |
| Fruits and vegetables | 76 | - | 0.420 | 0.500 | 0 | 1 |
| Egg or poultry | 76 | - | 0.070 | 0.250 | 0 | 1 |
| Meat | 76 | - | 0.200 | 0.400 | 0 | 1 |
| Milk and dairy | 76 | - | 0.080 | 0.270 | 0 | 1 |
| Honey | 76 | - | 0.110 | 0.310 | 0 | 1 |
| Bakery products | 76 | - | 0.260 | 0.160 | 0 | 1 |
| Herbs | 76 | - | 0.000 | 0.000 | 0 | 0 |
| Wine and grapes | 76 | - | 0.130 | 0.340 | 0 | 1 |
