# Supplementary material for: Farm diversification as a potential success factor for small-scale farmers constrained by COVID-related lockdown. Contributions from a survey conducted in four European countries during the first wave of COVID-19
Source: PLoS One. 2021 May 21;16(5):e0251715. doi: 10.1371/journal.pone.0251715 (PMC8139471; doi:10.1371/journal.pone.0251715)
Supplement: S4 Table — (DOCX) [file pone.0251715.s004.docx]

# S4 Table. Country-specific descriptive statistics (Romania).

| Variable | N | Frequency (%) | Mean | SD | Min | Max |
| --- | --- | --- | --- | --- | --- | --- |
| Increase in sales | 157 | - | 0.401 | 0.491 | 0 | 1 |
| Channel diversification | 157 | - | 4.847 | 3.653 | 0 | 12 |
| Number of channels: 0 | 18 | 11.5 | - | - | - | - |
| Number of channels: 1 | 15 | 9.6 | - | - | - | - |
| Number of channels: 2 | 18 | 11.4 | - | - | - | - |
| Number of channels: 3 | 17 | 10.8 | - | - | - | - |
| Number of channels: 4-6 | 41 | 26.1 | - | - | - | - |
| Number of channels: 7 or more | 48 | 30.6 | - | - | - | - |
| Product diversification | 157 | - | 1.636 | 1.321 | 1 | 9 |
| Number of products: 1 | 106 | 67.5 | - | - | - | - |
| Number of products: 2 | 29 | 18.5 | - | - | - | - |
| Number of products: 3 | 12 | 7.6 | - | - | - | - |
| Number of products: 4 | 5 | 3.2 | - | - | - | - |
| Number of products: 5 or more | 5 | 3.2 | - | - | - | - |
| Income | 157 | - | 2.439 | 1.226 | 1 | 5 |
| Below €5.000 | 38 | 24.2 | - | - | - | - |
| €5.000 - €15.000 | 56 | 35.7 | - | - | - | - |
| €15.000 - €30.000 | 35 | 22.3 | - | - | - | - |
| €30.000 - €50.000 | 12 | 7.6 | - | - | - | - |
| More than €50.000 | 16 | 10.2 | - | - | - | - |
| Fruits and vegetables | 157 | - | 0.460 | 0.500 | 0 | 1 |
| Egg or poultry | 157 | - | 0.140 | 0.350 | 0 | 1 |
| Meat | 157 | - | 0.180 | 0.380 | 0 | 1 |
| Milk and dairy | 157 | - | 0.340 | 0.470 | 0 | 1 |
| Honey | 157 | - | 0.120 | 0.330 | 0 | 1 |
| Bakery products | 157 | - | 0.070 | 0.260 | 0 | 1 |
| Herbs | 157 | - | 0.070 | 0.260 | 0 | 1 |
| Wine and grapes | 157 | - | 0.120 | 0.330 | 0 | 1 |
