# Supplementary material for: Farm diversification as a potential success factor for small-scale farmers constrained by COVID-related lockdown. Contributions from a survey conducted in four European countries during the first wave of COVID-19
Source: PLoS One. 2021 May 21;16(5):e0251715. doi: 10.1371/journal.pone.0251715 (PMC8139471; doi:10.1371/journal.pone.0251715)
Supplement: Attachment — Submitted filename: Response to Reviewers.docx [file pone.0251715.s006.docx]

**Response to the Reviewers' comments on “PONE-D-20-39115”**

**First, we thank the Reviewers for their comments. We believe that the paper has improved a lot. In the following pages we provide our reactions to the comments – the original comments are in standard text and un-edited, while our answers are marked in red. Lines refer to the line numbers of the “Revised Manuscript with Track Changes” document.**

**Reviewer #1**

I like the main idea of the article. It is original and contributes to the literature. However, the authors need to correct two basic issues:

- better explain the selection of the research sample and describe the farms they surveyed. Explain what was the method of sample/respondents selection.

**Thank you very much for directing our attention to this crucial issue. We would not like to claim that our data is representative of farms in the analysed countries – a lack of representativeness is typically the case when small-scale farms, or farmers participating in short food supply chains, are the focus of research. The most representative dataset would be that of the FADN, but this ignores truly small farms, and although we did make an attempt, it was basically impossible to have individual survey questions sent out to FADN farms. The manuscript has been supplemented to include a definition of small-scale farmers (lines 94-103), and a more detailed description of the sample selection process (lines 274-286).**

Why is the sample from Portugal so small?

**Our sample is composed of 52 Estonian, 136 Hungarian, 76 Portuguese, and 157 Romanian farms. Whilst we obviously aimed at including as large a sample size as possible, in practice, we needed to obtain access to farms, thus we needed the help of personal contacts (LEADER Local Action Group and NGO representatives, consumer purchase group organizers, etc.) for data collection. One should not forget that the survey was organized during the first wave of the pandemic, when farmers’ willingness to answer was very low because of their distress, but also because having face-to-face meetings with producers was impossible. Nonetheless, we believe that the high number of answers collected during the ongoing pandemic from countries with rather different production types ensure the robustness of our conclusions.**

The selection of the sample does not seem reliable and credible. How did you define small-scale farmers? What was a threshold to include a farm in the sample?

**Further sections have been added to the text about the definition of small-scale farmers (lines 94-103) and argumentation with respect to sample selection (lines 274-286). Instead of the use of arbitrary thresholds (noting that countries with rather different socio-economic contexts were involved in the analysis), small-scale farmers were defined through their participation in short food supply chains (as implied by Kneafsey et al., 2013, Martinez et al., 2010 and many others). Additionally, for convenience, farmers who cooperated with specific organizations (LEADER LAGs, NGOs, etc.) were regarded as small-scale ones. Limitations that arise in relation to this decision are now discussed in the revised text (lines 102-103 and 522-525).**

You write that “Relatively large small-scale farms are overrepresented in the Czech sample (farms with an annual income of 50,000 EUR or more make up about 52% of this specific subsample)”. I am confused not really sure how did you pick up your sample.

**Following your comments, we re-analyzed the Czech subsample. As these farms were mostly crop farms that did not place high importance on making sales through short food supply chains, we finally decided to remove them from the analysis. Thanks for this important remark. Although the remaining sample was also re-analyzed with this comment in mind, and was found to be adequate, this potential limitation has been discussed in the revised manuscript (lines 102-103 and 522-525).**

Are your farms really small-scale farms. Maybe provide more detailed sample statistic. Besides, is your sample anyhow related to FADN?

**More detailed descriptive statistics have been provided (S1, S2, S3 and S4 Tables). Our sample is not related to FADN, thus a brief note to that effect has been added (lines 342-345). Further elaboration of the sample selection process has been added to the revised manuscript and is explained above.**

If not, how did you reach these farms and selected them?

**Farms were reached through the representatives of LEADER Local Action Groups, NGOs, organizers of consumer purchase groups, etc. (See the comments above with the references to the new texts in the revised manuscript.)**

I suppose that your sample is far from being representative.

**You are right, our sample is not representative. A statement explicitly noting this has been added (lines 342-345); the limitations of FADN (not concerning small farms) and the need for new primary research and data collection have been discussed in the revised manuscript and are explained above.**

I am not even sure that it really includes only small-scale farms. In fact, I have no idea what farms you studied? Were they just small, how small, what was their production profile, with the addition of off-farm work, etc?

**You are right - this is why the Czech subsample was finally removed from the analysis. More detailed descriptive statistics (with information about production profiles) have been provided (S1, S2, S3 and S4 Tables). Off-farm work was included in the income variable, which in turn, was used as an indicator of farm size. (See also lines 304-305).**

- figures and tables are unclear and difficult to use/understand. The rule is that the table/figure should be easy to understand even without reading the text. Figures 1 and 2 are rather difficult to understand. Try to present the size structure in different way. Besides, the ranges are overlapping.

**Thank you very much for your comment, Figures 1 and 2 have been reorganized, and we hope they are now much more self-explanatory.**

Table 1 is also unclear. I understand that the column N is the number of farms. First of all, it should be noted that it is the number of holdings in the FADN system and that it is expressed in thousands. Secondly, for each type of production, the number of farms is the same, which obviously is a mistake.

**Thank you very much for pointing out this (now) obvious mistake. We have also clarified that in the case of cereal and other field crops, the unit is hectares, while for livestock farms the unit is total livestock units.**

Thirdly, in each column to a number of digits after dot should the same. And why are there only zeros in the minimum size of farm? The minimum economic size threshold for a farm to be included in FADN is 8 thousand euro, so it is impossible to have zero ha in case of every country - extend a number of digits after zero.

**Thanks for noting this – it was our mistake when calculating the summary statistics. (That’s also why country-specific N numbers were identical across production types). This has now been corrected.**

Data for Portugal seems unrealistic. Please double check. Figures 3 and 4 are also not very clear to me. Please describe the axes better.

**Thanks for raising your concern. Data for Portugal have been double-checked; they are correct. Please note, there are historical differences in the farm structures of post-communist economies (i.e. all other countries) and Portugal, where farms are predominantly family farms, with a much smaller size than those in the other sample countries. We have better emphasised the structural differences in the text, supported by newly added literature. We believe this difference actually adds to the robustness of our estimations.**

Some minor issues:

Line 158 – FADN does not use ESU anymore. The economic size of farm is currently measured by standard output (SO). Explain the AWU and how it differs among countries.

**Thank you, this has been corrected.**

Line 233 – You write “The structure of the dataset resulting from the interviews is as follows” but actually not table or information about data structure is following. Just some info how data was collected. This info is partly included in table 3, which is presented later on. But it is not enough.

**Actually, we meant the variables of the dataset: the text has been clarified, thanks for pointing out the discrepancy. With respect to Table 3 which presents the summary statistics of the variables employed, we have added country-specific information in S1, S2, S3 and S4 Tables of the Supplementary Material. In addition, following the comments of Reviewer #2, the distribution of frequencies have been presented for categorical variables.**

Line 251 – what do you understand by annual gross income – is it family income, farm income, agricultural income? Per person or total household. With off farm activities included?

**Thanks for pointing out this issue. The text has been rewritten. “…, producers were requested to identify their annual gross farm income (off-farm activities included, without government farm program payments)…”**

Line 338 – what was the estimation method? What kind of regression did you estimated. Was it a logistic regression?

**The text has been rewritten (lines 322-328) to clarify and to provide arguments in favour of the use of the semi-nonparametric (SNP) method defined by Gallant and Nychka (1987) as opposed to standard logit or probit estimations.**

English proof is required. Some minor language issues.

**The help of a professional native-English-speaking proof-reader was employed**

Overall, pay attention to details and try to explain details better.

**We hope this revised version of our submission will meet these expectations.**

**Reviewer #2:**

General comments:

The manuscript is very well written and organized. Although the manuscript is not exceptionally evolved in methodological terms or in the results obtained, it discusses a relevant topic in the pandemic context, allowing to bring to light some data obtained through surveys of small farmers.

These two specific contexts (that of the pandemic and that of small farmers) are relevant, the first of which is shrouded in uncertainty and can be characterized by the need for constant reaction to policy measures that change at a breakneck pace, and the second still it is insufficiently studied in Europe, particularly with regard to its importance in the economy and in rural society as a whole. I recommend to the authors some of the recent papers that have been published in the framework of the European SALSA project (mainly that of Rivera et al., I think it may reinforce the discussion and/or the introductory sections a little more) (<https://doi.org/10.1016/j.gfs.2020.100395>; <https://doi.org/10.1016/j.gfs.2020.100389>; <https://doi.org/10.1016/j.gfs.2020.100417>; <https://doi.org/10.1016/j.gfs.2020.100416>; <https://doi.org/10.1016/j.gfs.2020.100412>; <https://doi.org/10.1016/j.gfs.2020.100425>; <https://doi.org/10.1016/j.gfs.2020.100427>). The sub-section “Specificities of the countries involved in the study” can be also strengthen through the literature. I recommend the paper “Typology and distribution of small farms in Europe: Towards a better picture” (<https://doi.org/10.1016/j.landusepol.2018.04.012>) and references therein. Therefore, I recommend the manuscript for publication after minor revision.

**Thank you for your guidance: the proposed literature is indeed very useful, we have studied it carefully and referenced it in the revised manuscript. New content has been developed and included with respect to the comparison of the countries that are included, the specificities of small farms, problems with defining small-scale farmers, etc. (Lines 94-103; 118-125; 207-215, etc.)**

Detailed suggestions:

L25: Change “As many as 19 percent” to “Approximately 19%” or “About 19%”

**Thank you. Suggestion accepted.**

L42-43: Add references.

**References have been added.**

L43: Remove “In order”

**We have removed it.**

L46: Change “On the other hand, lockdown measures meant that” to “Lockdown measures also meant that”

**Thank you. Suggestion accepted.**

L53: Change “In this paper, the focus is on farmers who” to “In this paper, we focused on the farmers who”

**Thank you. Suggestion accepted.**

L101: Change “On the other hand” to “Moreover”

**Thank you. Suggestion accepted.**

L104-105: Please clarify. As you start started the sentence with "In this paper ..." it was not clear whether you refer to your own manuscript or to the paper you just cited in the previous sentence (Hunt et al., 2012)

**Thank you. Clarified.**

L134: Remove “in order”

**Thank you. Suggestion accepted.**

L135: Remove “business”

**Thank you. Suggestion accepted.**

L149: Remove “Data from countries with very different characteristics were used in the analysis”. This sentence adds nothing.

**The text has been changed: “To increase the relevance and robustness of the results, data from countries with very different characteristics were used in the analysis.”**

L233: You refer only Hungary. And the remaining countries?

**The text has been amended. “The Ethics Review Procedure complied with the Guidance Note “Ethics in Social Sciences and Humanities” issued by the European Commission in 2018. The questionnaire, the use of verbal consent, and other ethical aspects were approved by the Ethical Committee of the Centre for Economic and Regional Studies, Hungary – the lead country of this research. The questionnaire was translated and used to collect data in other countries (all of them part of the European Union).”**

L255: Remove “in order”

**Suggestion accepted.**

L276: Remove “In order”

**Suggestion accepted.**

L286: Regarding Table 3, it seems to me to make more sense to present the distribution of frequencies for categorical variables than the mean and standard deviation (and by country).

**More detailed descriptive statistics have been provided (S1, S2, S3 and S4 Tables) with the distribution of frequencies for categorical variables.**

**References**

**Gallant AR, Nychka DW. Semi-nonparametric maximum likelihood estimation. Econometrica: Journal of the econometric society. 1987:363-90.**

**Kneafsey M, Venn L, Schmutz U, Balázs B, Trenchard L, Eyden-Wood T, et al. Short food supply chains and local food systems in the EU. A state of play of their socio-economic characteristics. JRC scientific and policy reports. 2013;123:129.**

**Martinez S, Hand M, Da Pra M, Pollack S, Ralston K, Smith T, et al. Local food systems: concepts, impacts, and issues. University Library of Munich, Germany, 2010.**
