# Supplementary material for: Farm diversification as a potential success factor for small-scale farmers constrained by COVID-related lockdown. Contributions from a survey conducted in four European countries during the first wave of COVID-19
Source: PLoS One. 2021 May 21;16(5):e0251715. doi: 10.1371/journal.pone.0251715 (PMC8139471; doi:10.1371/journal.pone.0251715)
Supplement: Attachment — Submitted filename: Response to Reviewers.docx [file pone.0251715.s007.docx]

**Response to the Reviewer's comments on “PONE-D-20-39115_R1-1”**

**First, we thank the comments and the detailed guidance provided by Reviewer #2. We hope that we will fully meet PLOS ONE’s publication criteria with the current revision.**

**In the following pages we provide our reactions to the comments – the original comments are in standard text and un-edited, while our answers are marked in red.**

**Additionally, the reference list has been extended by inserting three further references ([49-51] in the revised manuscript) in order to follow the Reviewer’s suggestion concerning a brief explanation of the LEADER LAGs and their role in organizing small-scale farmers, to account for non-European readers.**

**Thank you for the possibility for revising our manuscript.**

**Reviewer #2:**

Regarding the response to the comments I made in the first review, there is nothing to add, the authors complied and responded accordingly. However, I have carefully read the comments of Reviewer # 1, which I consider relevant and important, and therefore deserve special attention by the authors. It is very difficult to achieve good levels of representativeness when the focus of the analysis is on small farms, because there is a lack of knowledge about their real diversity. This fact, in itself, makes the publication of this manuscript important, which relevance is exacerbated by the pandemic context in which we live. Furthermore, the authors never aspired to achieve this representativeness, and this seems clear to me in the manuscript. The issue related to the size of farms can be better explained, and some of my suggestions go in this direction. In my opinion, these issues can be overcome through minor adjustments to the manuscript, so they do not jeopardize its publication, the general objectives, and the analytical process.

**Thank you for your careful consideration.**

Title

I think that the authors should change the title. The authors did not determine the "ideal level", for that you would need other statistical procedures, and a larger and more diverse sample. I think it would be more adjusted to the manuscript content to reformulate the first part of the title as a question. The second part of the title (“An international study”), in addition to being unappealing, creates expectations in the reader that can then be frustrated. Thus, my proposal is:

Is farm diversification a success factor for small-scale farmers constrained by COVID-related lockdown? Contributions from a survey conducted in four European countries during the first wave of COVID-19

**Thank you. Your suggestion has been accepted, though a slight modification has been made in order to comply with the authors’ guidelines (as to titles should be written in a sentence case). Thus, the rewritten title reads as “Farm diversification as a potential success factor for small-scale farmers constrained by COVID-related lockdown. Contributions from a survey conducted in four European countries during the first wave of COVID-19”.**

Introduction (and related 3 sub-sections)

L48-49: Suggestion: Change “The virus, simultaneously causing global and local economic as well as social disturbances [11], (…)” to “The virus, while simultaneously causing economic and social disturbances at multiple scales [11], (…)”

**Thank you. Suggestion accepted.**

L51: Move the sentence "Small farms have been (...)" to a new paragraph

**Thank you. Suggestion accepted.**

L68: Consider change the title of the sub-section to “Linkages between Short Food Supply Chains and small-scale farmers”

**Thank you. Suggestion accepted.**

L69-70: Simply stating that the interest remains “undiminished” is not enlightening. The reader will remain unsure whether the interest is low or high.

**You are right, thank you. The revised text reads as “Consumer interest in local food remains high…”**

L70-72: Reformulate the sentence. Suggestion: “Despite the growing attention of researchers and policy makers to local food systems, alternative food networks and short food supply chains, their respective definitions remains unclear.”

**Thank you. Suggestion accepted.**

L72-75: Change the sentence as follows (suggestion): “In our study we followed the approach of Gruchmann et al. [18] and Schmutz et al. [17], mainly focusing on producer-consumer interactions involving producers directly selling their products to consumers, or through a limited number (ideally, zero) of intermediaries.”

**Thank you. Suggestion accepted.**

L78: Change “(…) many pieces of national legislation [19] (…)” to “(…) several legislative instruments [e.g., 19] (…)”

**Thank you. Suggestion accepted.**

L87-93: Rephrase as follows (suggestion): “As above mentioned for short food supply chains, also defining small-scale farmers is challenging [14, 23] and it is often based on certain thresholds that are highly dependent on the geographical context of the analysis [14]. Since the countries involved in our study represent markedly different contexts [14, 24] and building on the considerations of Kneafsey et al. [21] and Martinez et al. [22], small-scale farmers were identified through their participation in short food supply chains, instead of using a specific threshold.”

**Thank you. Suggestion accepted.**

L93-96: Move this sentence to the Material and Methods section.

**Thank you. Suggestion accepted.**

L161-251: The subsection "Specificities of the countries involved in the study" should be moved to the Results’ section. The authors explore statistical data that characterize the target-countries of this manuscript, and discuss their differences and specificities. It does not seem to me an introductory section built on the basis of the state of the art, but results. If this option is followed by the authors, they should separate the methodological elements and integrate them in the Materials and Methods section.

**You are right. Suggestion accepted, thank you.**

L162-163: Remove the first sentence. The idea is repeated in the third sentence of this paragraph.

**Thank you for raising awareness; the third sentence of the paragraph has been removed.**

L177-179: Remove parentheses

**Thank you. Suggestion accepted.**

L200-201: Remove “In an excellent review,“ and change “finds” to “observed” (or to “found”, in alternative).

**Thank you. Suggestions accepted.**

L210: Change “The average size of a cereal farm is greatest in Estonia, where the latter are 20 times larger than their Portuguese counterparts” to “The largest mean cereal farm size can be found in Estonia, about 20 times larger than the Portuguese value, the lowest one, according to the data from FADN”. Additionally, at first reading the distribution of this values (Table 1) seems awkward, because they seem to contradict the majority of scientific publications on these topics. However, the authors must reinforce that this values are related to “production mix” farms, and clarify and discuss the rationale behind this option (most likely, this nuance was not clear to Reviewer #1, which led him to question the reliability of this data).

**Thank you for this comment. We have changed the text accordingly. With respect to the second suggestion, we realised the term “production mix” might have been misleading. We actually used the supposedly representative European farm structure database, the FADN to emphasise farm size differences in selected agricultural sectors (cereals, other field crops and animal husbandry) of the countries in our sample. (Thus, the data in Table 1 is fully in line with conclusions of the literature you have so kindly recommended us). We have rewritten the text, to better reason Table 1.**

L211: Change “picture” to “distribution”

**Thank you. Suggestion accepted.**

L231: Remove “banned”

**Thank you. Suggestion accepted.**

L233-236: Rephrase as follows: “Telework was centrally mandated for some professionals, such as civil servants in Portugal, but many more decided to stay at home in all countries, either to supervise their children (as the institutions of education had closed everywhere), making the use of the home office as a general rule.”

**Thank you. Suggestion accepted.**

L236: Change “Though” to another synonym to avoid two sentences in the same paragraph starting with the same word.

**Thank you. Suggestion accepted.**

L255: Change “help” to “contribution”

**Thank you. Suggestion accepted.**

Material and methods

L262: Move the sentence from L93-96 to this point. Additionally, for non-European readers, the authors must clarify what the LEADER Local Action Groups are, and their relation with small farming, so that there is no doubt that your sample is made up of small farmers. This doubt of Reviewer #1 seems to me not to be completely clarified in this version of the manuscript.

**Thank you for raising our awareness. Suggestions have been accepted; the manuscript has been rewritten to incorporate a short description of the LEADER LAGS and their role in the organization of local food systems and the coordination of small-scale farmers.**

L263: Move to another paragraph.

**Thank you. Suggestion accepted.**

L272-273: Change as follows: “Concerning the product categories commercialized by each farmer, data were coded into dummies: “1” if yes, “0” otherwise.”

**Thank you. Suggestion accepted.**

L282: Change “calculations” to “estimations”

**Thank you. Suggestion accepted.**

L302: Change “explanatories” to “explanatory variables”

**Thank you. Suggestion accepted.**

L305-306: Remove “Thus, the straightforward option for model estimation would be a logit or probit model.” You did not use any of these, why mention?

**Thank you. Suggestion accepted.**

L306-309: Rephrase as follows: We used the semi-nonparametric (SNP) method defined by Gallant and Nychka [54] due to its robustness when compared to the standard models.” and I think you can remove this part “(…) – furthermore, a post-estimation test of the null hypothesis whether a probit estimation would suffice, was considered easy to conduct.”

**Thank you. Suggestions accepted.**

L310-311: Change “(…) non-parametric approach is that, unlike parametric estimators, it is not sensitive to departures from distributional assumptions (…)” to “non-parametric approach is that, unlike parametric estimators, it is not sensitive to distributional assumptions (….)”.

**Thank you. Suggestion accepted.**

Results and discussion

L334: Change “earlier” to “previous”

**Thank you. Suggestion accepted.**

L335: Change “tended” to “tend” and “market” to “sell”

**Thank you. Suggestions accepted.**

L336-338: Reformulate this sentence to increase readability

**Thank you. The revised text reads as “Only those product categories are displayed in Table 3 the importance of which exceeded ten percent across the whole sample”.**

L365: Change “appeared” to “seems”

**Thank you. Suggestions accepted.**

L390: I think you need to add an additional measure of performance of each model (an adjusted R2; or the deviance D2).

**We most certainly would have added fit statistics, but nonparametric and semi-nonparametric models do not have R2 or D2 measures of fit. In the previous versions of the paper we were contemplating the idea of presenting the basic Probit Pseudo R2 as a guidance (that is equivalent when SNP is estimated with order 1 Hermite polynomials). And since the adequacy of Probit was rejected in all cases in favour of SNP models, it would follow, those statistics are minimum performance measures for our models. But the explanation (as you observe here) is a bit cumbersome and since it might only confuse readers in exchange of little added value, we removed them. (For your information, these are: M1 Pseudo R2 is 0.19; for M2 is 0.16 and finally, for M3 is 0.21).**
